# Longitudinal Gut Bacterial Colonization and Its Influencing Factors of Low Birth Weight Infants During the First 3 Months of Life

**DOI:** 10.3389/fmicb.2019.01105

**Published:** 2019-05-15

**Authors:** Cheng Chi, Yong Xue, Na Lv, Yanan Hao, Ruixia Liu, Yanxin Wang, Xin Ding, Huihui Zeng, Geng Li, Qun Shen, Xiaosong Hu, Lijun Chen, Tiemin Jiang, Junying Zhao, Nicholas Buys, Jing Sun, Chenghong Yin, Baoli Zhu

**Affiliations:** ^1^Department of Internal Medicine, Beijing Obstetrics and Gynecology Hospital, Capital Medical University, Beijing, China; ^2^College of Food Science and Nutritional Engineering, China Agricultural University, Beijing, China; ^3^CAS Key Laboratory of Pathogenic Microbiology and Immunology, Institute of Microbiology, Chinese Academy of Sciences, Beijing, China; ^4^Department of Central Laboratory, Beijing Obstetrics and Gynecology Hospital, Capital Medical University, Beijing, China; ^5^Department of Obstetrics, Beijing Obstetrics and Gynecology Hospital, Capital Medical University, Beijing, China; ^6^Department of Neonatology, Beijing Obstetrics and Gynecology Hospital, Capital Medical University, Beijing, China; ^7^National Engineering Center of Dairy for Early Life Health, Beijing Sanyuan Foods Co., Ltd., Beijing, China; ^8^School of Medicine, Griffith University, Brisbane, QLD, Australia; ^9^Beijing Key Laboratory of Microbial Drug Resistance and Resistome, Beijing, China; ^10^Collaborative Innovation Center for Diagnosis and Treatment of Infectious Diseases, The First Affiliated Hospital, College of Medicine, Zhejiang University, Hangzhou, China; ^11^Department of Pathogenic Biology, School of Basic Medical Sciences, Southwest Medical University, Chengdu, China

**Keywords:** low birth weight infant, gut microbiota, intestinal colonization, microbial diversity, influencing factor

## Abstract

Establishment of low birth weight (LBW) infant gut microbiota may have lifelong implications for the health of individuals. However, no longitudinal cohort studies have been conducted to characterize the gut microbial profiles of LBW infants and their influencing factors. Our objective was to understand how the gut bacterial community structure of LBW and normal birth weight (NBW) infants varies across the first 3 months of life and assess the influencing factors. In this observational cohort study, gut bacterial composition was identified with sequencing of the 16S rRNA gene in fecal samples of 69 LBW infants and 65 NBW controls at 0 day, 3 days, 2 weeks, 6 weeks, and 3 months (defined as stages 1–5) after birth. Alpha-diversity of both groups displayed a decreasing trend followed by slight variations. There were significant differences on the Shannon index of the two groups at stages 1 to 3 (*P* = 0.041, *P* = 0.032, and *P* = 0.014, respectively). The microbiota community structure of LBW infants were significantly different from NBW infants throughout the 3 months (all *P* < 0.05) but not at stage 2 (*P* = 0.054). There was a significant increase in abundance in Firmicutes while a decrease in Proteobacteria, and at genus level the abundance of *Enterococcus*, *Klebsiella*, and *Streptococcus* increased while it decreased for *Haemophilus* in LBW group. Birth weight was the main factor explaining the observed variation at all stages, except at stage 2. Delivery mode (4.78%) and antibiotic usage (3.50%) contributed to explain the observed variation at stage 3, and pregestational BMI (4.61%) partially explained the observed variation at stage 4. In conclusion, gut microbial communities differed in NBW and LBW infants from birth to 3 months of life, and were affected by birth weight, delivery mode, antibiotic treatment, and pregestational BMI.

## Introduction

Low birth weight, defined as less than 2,500 × *g*, is a common but serious event, despite improvements in healthcare (Franz et al., 2018). The prevalence of LBW has increased in recent years, occurring in 15% of births worldwide (Luyckx et al., 2017), in part due to increases in multiple pregnancies and *in vitro* fertilization. It is acknowledged that LBW infants have an immature gastrointestinal tract (GIT) and immune system and their diminished gut barrier allows for translocation of bacteria from the gut to the bloodstream, leading to systemic inflammation, which puts them at high risk for diseases associated with high morbidity and mortality rates (Moschopoulos et al., 2018; Wisgrill et al., 2018).

The importance of gut microbiota in the LBW infants is clearly evident in considering the risks of developing late-onset sepsis (Keunen et al., 2015) and necrotizing enterocolitis (NEC) (O’Connor et al., 2016). Furthermore, altered gut microbiota has been reported in children with diseases such as autism (Neu and Walker, 2011), diabetes (Khashan et al., 2015) and cerebral palsy (Cong et al., 2015), which represents a major cost to health-care services and society due to lifelong physical and mental impairment. The gut microbiota of infants undergoes rapid dynamic changes in the first few months to years of life and these changes are hypothesized to affect their health (Madan et al., 2012; Carlson et al., 2018). LBW infants have distinct gut microbiota with lower diversity and greater abundance of potential pathogens (Brooks et al., 2018) such as *Enterococcus* spp., *Staphylococcus aureus*, *Klebsiella* spp., *Acinetobacter* spp., *Pseudomonas aeruginosa*, and *Enterobacteriaceae*, which are also the most frequent cause of nosocomial infections (Hu et al., 2015). However, less research has been done on the gut bacterial colonization progress of LBW infants during the early postnatal period, especially in urban Chinese infants. Such research is important as the ethnic origin of individuals may be a key factor to consider in microbiota research (Deschasaux et al., 2018).

The factors responsible for the altered microbial colonization patterns observed in LBW infants compared to their NBW counterparts are not well-defined. Previous studies revealed that the microbial colonization process may be initiated prenatally by a distinct microbiota in the placenta and amniotic fluid, with the microbiota of meconium affected by maternal factors (Neu and Walker, 2011). After birth, most LBW infants stay in hospital to receive observation and treatment for at least 1–2 weeks. A higher incidence of opportunistic infections in LBW infants increases their exposure to antibiotics, profoundly affecting their gut microbiota (Arias and Murray, 2012). In conjunction with their immaturity, factors such as cesarean (C-) section, small size for gestational age, prolonged exposure to hospital environments, and less breastfeeding were also likely contributors to aberrant gut bacterial colonization (Bentley et al., 2016; Arboleya et al., 2017; Grier et al., 2017).

Prior to our research, no study had been conducted to characterize the gut microbial profile of Chinese LBW infants and its influencing factors. In this prospective, observational cohort study, we aimed to evaluate how gut bacterial community structure of LBW and NBW infants varies across the 3 months of early life and assess the effects of various exposures on gut microbiota, such as birth weight, delivery mode, hospital stay, antibiotic administration and feeding practices.

## Materials and Methods

### Study Population and Fecal Sample Collection

The study was approved by the Ethics Committee of the Beijing Obstetrics and Gynecology Hospital (No. 2017-KY-027-01), and informed written consent was obtained from parents of each infant. The study was registered in Chinese Clinical Trials Register (registration number ChiCTR1800014390) on January 10, 2018. Sixty-nine LBW infants (birth weight < 2,500 g) and 65 NBW (birth weight ≥ 2,500 g) infants were enrolled in this prospective observational cohort study and the inclusion criteria were as follows: All the pregnant women were admitted to the Beijing Obstetrics and Gynecology Hospital before delivery, the LBW infants were immediately hospitalized in the Neonatal Intensive Care Unit (NICU) of the Beijing Obstetrics and Gynecology Hospital after delivery, and the NBW infants were immediately placed in the newborn nursery with their mother after delivery. Infants were excluded if they had neonatal asphyxia, congenital heart disease, neural tube defects, genetic defects, or had diseases requiring surgery during the 3-month follow-up period. Fecal samples were collected by using a collection tube pre-filled with 8 mL of DNA stabilizer solution (PSP^^®^^ Spin Stool DNA Plus Kit, STRATEC Biomedical AG, Birkenfeld, Germany) from soiled diapers, which could allow collection, transport, and storage of the samples without DNA degradation for 3 months at RT. The fecal samples were delivered immediately to the laboratory and then stored at −80°C until DNA extraction. Fecal samples were collected at 0 day, 3 days, 2 weeks, 6 weeks, and 3 months post-birth, periods that were defined, respectively, as Stage 1, Stage 2, Stage 3, Stage 4, and Stage 5. NBW infants in the newborn nursery were accompanied by their mothers, fed with breast milk during the first 2–3 days after birth, and then discharged. In contrast, the LBW infants were immediately hospitalized in the NICU after their birth to receive observation and treatment for at least 1–2 weeks, during which time parental skin-to-skin contact was impossible. However, breast milk from their mother was collected and provided to the LBW infants or supplemented by infant formula as a nutritional alternative. In our study, 40.6% LBW infants (28/69) were hospitalized over 2 weeks after their birth. Information about their mothers was collected before delivery including age, height, pre-pregnancy weight, and history of GDM and gestational hypertension. Additionally, information on delivery mode, gestational weeks, gender and birth weight of infants was recorded at delivery. Information on antibiotic usage and length of hospital stay were recorded from medical records and type of feeding was collected by telephone follow-up at each of the stages following discharge.

### High Throughput 16S rRNA Amplicon Sequencing

Microbial DNA was isolated from fecal samples using the PSP Spin Stool DNA Plus kit (PSP^^®^^ Spin Stool DNA Plus Kit, STRATEC Biomedical AG, Birkenfeld, Germany), following the manufacturer’s instructions, which included a bead-beater step. The V4 hypervariable region of the 16S rRNA gene were amplified, sequenced and analyzed to define composition of the bacterial community. Amplification primers were generated with the following PCR primers: Forward, 515F (5′-GTGCCAGCMGCCGCGGTAA-3′); reverse, 806R (5′-GGACTACHVGGGTWTCTAAT-3′). The first PCR conditions were as follows: initial denaturation at 95°C for 3 min, followed by 25 cycles of 95°C for 30 s, 55°C for 30 s and 72°C for 30 s, followed by a final extension at 72°C for 5 min and then holding at 4°C. The first PCR products were excised from 1% agarose gel electrophoresis and purified using AMPure XP beads, followed by index PCR. Each index PCR reaction mixture contained the purified products of the first PCR experiment (5 μl), 5μl of both Nextera XT index primer 1 and primer 2, and 1X KAPA HiFi Hotstart Ready Mix. The second PCR conditions were as follows: 95°C for 3 min, followed by eight cycles of 95°C for 30 s, 55°C for 30 s and 72°C for 30 s, and finally, 72°C for 5 min. The index PCR products were purified with AMPure XP beads and the final amplicon libraries were approximately 630 bp. After pooling equally with unique indices, the multiplex amplified libraries were denatured with NaOH and sequenced by Illumina MiSeq with 2 × 300 paired-end V4 sequencing reagents (Illumina, San Diego, CA, United States). Sequence data were deposited in the NCBI Sequence Read Archive (SRA) under BioProject PRJNA 503135.

### Data Analysis

All paired-end reads from the original DNA fragment were assigned to each sample according to the unique barcodes and then merged using FLASH (Magoč and Salzberg, 2011). Merged sequences of poor quality (base calling errors, small insertions/deletions) were identified and removed by QIIME (Quantitative Insights into Microbial Ecology, version 1.9.1) quality filters under specific filtering conditions (*Q*-score ≥ 25). Chimera removal and dereplication of the reads was performed by using USEARCH8 (Edgar, 2010). The total high-quality merged sequences of each sample were classified into unique sequences after removing redundancies, and unique sequences with ≥97% sequence similarity were assigned to the same operational taxonomic units (OTUs). A representative sequence was selected for each OTU and assigned to different genera by the Classifier approach (Wang et al., 2007) in the Ribosomal Database Project (RDP) database (Cole et al., 2009). Alpha-diversity indices (bacterial richness: Chao1 index; bacterial diversity: Shannon index) based on the OTU level abundance profile were calculated using the Vegan package in R software (version 3.5.1) and compared by using Wilcoxon Rank sum test. Phylogenetic measures of beta-diversity based on the genus level abundance profile were also calculated by using the Vegan package, and PCoA plot based on Bray–Curtis distances were performed using the ggplot2 package. The top two principal coordinates (PC1 and PC2, representing the maximum amount of variation present in the dataset) were compared in each group. The adonis permutational multivariate analysis of variance (PERMANOVA) of Bray–Curtis distances with 9,999 permutations was used to compare the microbiota community structure across stages and groups. Differential abundance of genera and phyla were examined using the Wilcoxon rank sum test, and *P*-values were corrected for multiple testing using the Benjamin and Hochberg method. Linear discriminant analysis (LDA) of effect size (LEfSe) was applied to determine the most discriminant taxa between NBW and LBW groups at week 2 and 6 after birth (LDA score ≥ 3.0 and *P*-value < 0.05), and then the canonical RDA was conducted with the Vegan package to better understand the relationship between gut microbial composition and characteristics of mothers and infants. R software was used for plotting and statistical analysis.

## Results

### Study Cohort

**FIGURE 1:**
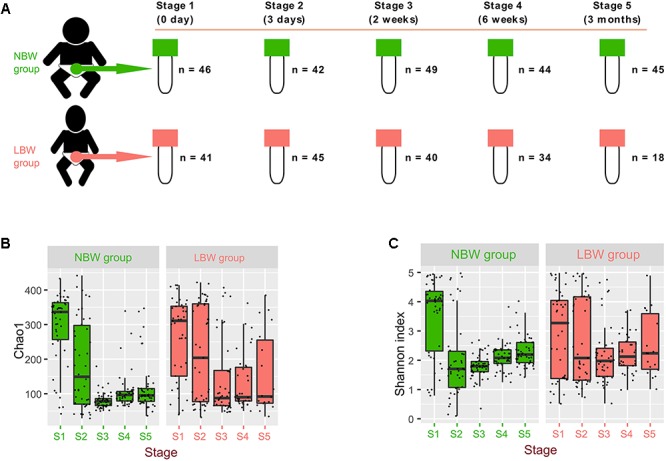
Microbiota diversity varied by birth weight and sampling time. Total 404 samples were collected from 134 infants at 0 day, 3 days, 2 weeks, 6 weeks, and 3 months after their birth (defined as different stages range from stage 1 to stage 5) **(A)**. Alpha-diversity of gut microbiota, measured by Shannon **(B)** and Chao1 **(C)** index.

A total of 404 samples from 0 day (meconium), 3 days, 2 weeks, 6 weeks, and 3 months after birth were obtained from 134 infants (65 were allocated to the NBW group and 69 to the LBW group based on their birth weight) selected in this cohort study (Figure 1A). Mothers of LBW infants did not differ in age or pregestational BMI from mothers of NBW infants but they were more likely to be diagnosed with GDM and gestational hypertension. LBW infants had lower gestational age at birth and they were more often born by C-section and exposed to antibiotics, and they were introduced to formula due to a lack of breast milk in the early postnatal period (Table 1 and Supplementary Table 1).

**Table 1:** Characteristics of the study participants.

	NBW (*n* = 65)	LBW (*n* = 69)	*P*
Gestational age, weeks (mean, *SD*)	38.4 (1.2)	34.6 (3.0)	<0.001
Birth weight, g (mean, *SD*)	3261.5 (336.3)	2063.5 (425.4)	<0.001
C-section birth (*N*, %)	37 (56.9)	54 (78.3)	0.003
Male (*N*, %)	32 (49.2)	31 (44.9)	0.618
Antibiotics at stage 3 (*N*, %)	4 (6.2)	37 (53.6)	<0.001
Hospital stay at stage 3 (*N*, %)	0 (0)	28 (40.6)	<0.001
Type of feeding at stage 3 (*N*, %)^∗^			<0.001
Exclusive breastfeeding	40 (64.5)	10 (16.4)	
Mixed feeding	22 (35.5)	48 (78.7)	
Formula	0 (0)	3 (4.9)	
Maternal			
Age (mean, *SD*)	33.3 (4.4)	32.0 (3.6)	0.056
GDM (*N*, %)	6 (9.2)	15 (21.7)	0.047
Gestational hypertension (*N*, %)	1 (1.5)	14 (20.3)	<0.001
Pregestational BMI (mean, *SD*)^∗^	22.3 (2.8)	22.2 (4.2)	0.727

*^∗^Missing data: Type of feeding at stage 3 (n = 11); pregestational BMI (n = 16).*

### Microbiota Diversity and Composition Varied by Birth Weight and Sampling Time

Our results showed that alpha-diversity (Shannon and Chao1 index) of gut microbiota (Supplementary Table 2 and Figure 1B,C), firstly decreased and then varied slightly in the NBW group. However, the Chao1 and Shannon indices in the LBW group were stable, except for the comparison of the Chao1 index between stage 2 and stage 3 (*P* = 0.032). Additionally, there were no significant differences of the Chao1 index between the two groups at corresponding stages (Supplementary Table 3), except at stage 3 (*P* < 0.001). Interestingly, the Shannon index of the NBW group was significantly higher than it was in the LBW group at stage 1 (*P* = 0.041) and lower in stages 2 and 3 (*P* = 0.032 and *P* = 0.014, respectively).

**FIGURE 2:**
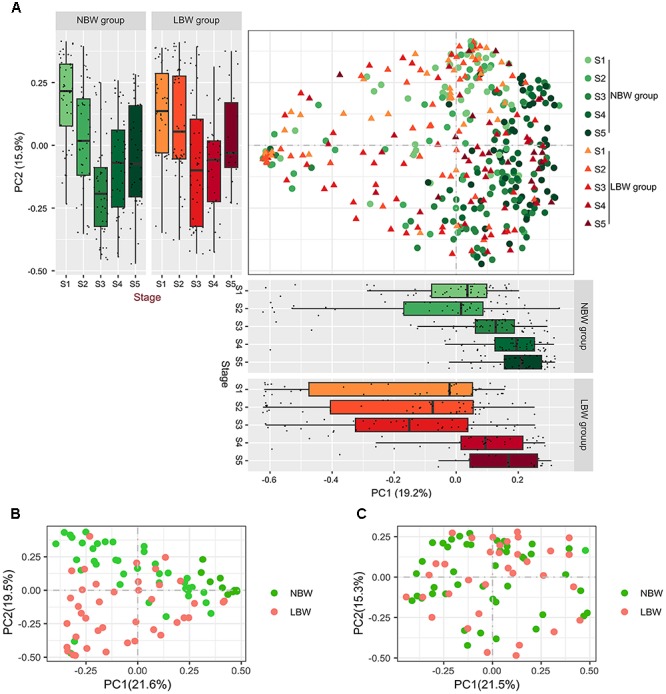
Gut bacterial community structure between LBW and NBW groups. PCoA **(A)** of Bray–Curtis distances based on the profile of OTU: each dot represents a sample and each color indicates a time point. The distribution of samples by body site is shown along the first and second axes of the PCoA plot. PCoA of microbial communities between LBW and NBW groups at stage 3 **(B)** and stage 4 **(C)**. PCoA, principal coordinate analysis; LBW, low birth weight; NBW, normal birth weight.

We used PCoA based on Bray–Curtis dissimilarity to examine the relationship of microbial communities across different groups and stages (Figure 2A). Bacterial compositions of samples showed a change trend over time among the five stages, showing that samples from both NBW and LBW infants at the initial stages (shown in light color) tended to locate in the left of the graph, when compared to the samples in the later stages (shown in dark color), which located in the right of the graph. The distribution of samples by stages and groups is shown along the first and second axes of the PCoA plot. Along the first axis, the value of PC1 in NBW infants showed an increasing trend over time, similar with LBW infants. However, PC1 of LBW infants was significantly reduced at stage 3 when compared with the other stages (all *P* < 0.01). Along the second axis, sample distribution of the two groups showed a similar change trend over time in PC2. We compared the β-diversity distances within and between the groups to see whether there are distinctive associations. Microbiota community structure of NBW infants differed over time in the first 6 weeks of early life, when comparing different time points across all samples in the same group (all adonis *P* < 0.05). Though similar differences were also found in LBW infants, there was no significant difference in microbiota community structure between stages 1 and 2 (adonis *P* = 0.204). Furthermore, no significant differences of microbiota community structure between stages 4 and 5 were found in NBW and LBW infants (adonis *P* = 0.504 and *P* = 0.254, respectively). We also found the microbiota community structure of NBW infants was significantly different from that of LBW infants (Supplementary Table 4) at stages 1, 3, 4, and 5 (all adonis *P* < 0.05) but not at stage 2 (adonis *P* = 0.054). In our cohort, 4.6% infants (3/65) in the NBW group was preterm infants, and 27.5% infants (19/69) in the LBW group was term infants. To address the possible influence of gestational age, we added a subgroup analysis of the difference of the microbiota community structure between term NBW and preterm LBW infants (Supplementary Table 5). The microbiota community structure of term NBW infants were significantly different from that of preterm LBW infants at stages 1, 3, 4, and 5 (all adonis *P* < 0.05) but not at stage 2 (adonis *P* = 0.176). Besides, no significant difference was observed in the microbiota community structure between the samples of preterm LBW and term LBW infants (adonis *P* > 0.05).

Considering the fact that microbiota community structure of LBW infants was significantly different from NBW infants at stages 3 and 4, we further explored bacterial community structure at these two stages. The results of plotting the infant microbiota of stage 3 showed obviously that the microbiota tended to cluster on a basis of birth weight. It is clear that spots separated well between two groups and clustered together in the same group at stage 3 (Figure 2B), indicating the existence of marked bacterial community structural differences between the two groups (adonis *P* < 0.001). Similarly, differences in the bacterial community structure between two groups was also found at stage 4 (adonis *P* = 0.006), although it was not obvious in PCoA plot (Figure 2C).

**FIGURE 3:**
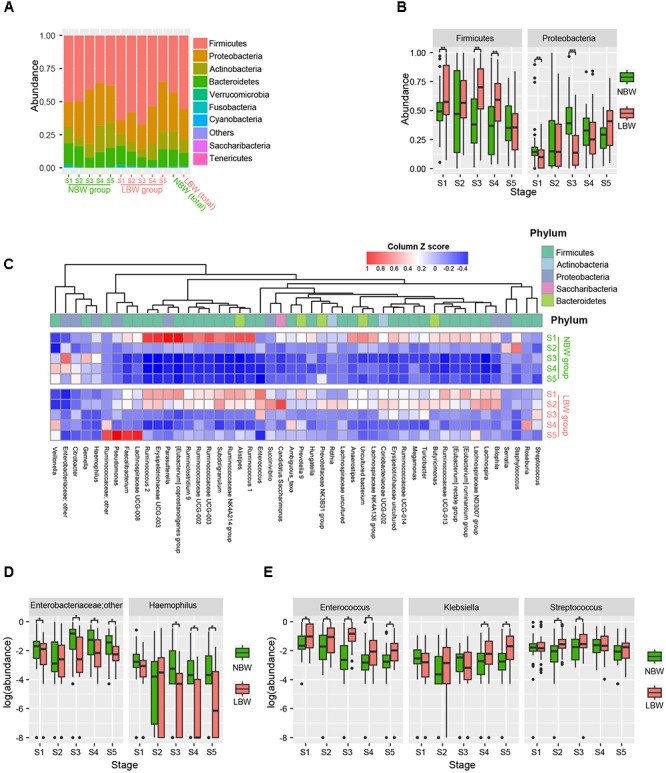
Phyla and genera strikingly different between the LBW and NBW groups. Phylum level gut microbial relative abundance at all stages **(A)**. Relative abundance of gut microbiota at phylum level, including Firmicutes and Proteobacteria **(B)**. Differentiated genera between LBW and NBW groups were displayed by using heatmap according to different stages and groups (adjust *P* < 0.01, Wilcoxon rank sum test corrected for multiple testing by the Benjamini and Hochberg method), and the abundance profiles are transformed into *Z*-scores by subtracting the average abundance and dividing the standard deviation of all samples **(C)**. Relative abundance of gut microbial taxa at genus level, including an unknown *Enterobacteriaceae* genus and *Haemophilus* enriched in NBW group **(D)**, and *Enterococcus*, *Klebsiella*, *Streptococcus* enriched in LBW group **(E)**. LBW, low birth weight; NBW, normal birth weight. ^∗^*P* < 0.05, ^∗∗^*P* < 0.01, ^∗∗∗^*P* < 0.001.

The dominant bacteria identified in LBW infant stool samples (Figure 3A) was Firmicutes, making up 55.5% of normalized reads, followed by phylum Proteobacteria (23.2%), Bacteroidetes (10.2%), and Actinobacteria (9.8%). In the NBW group, Firmicutes was also the most common phylum, making up 42.9% of all normalized reads, followed by Proteobacteria (29.4%), Actinobacteria (13.8%), and Bacteroidetes (12.9%). We found lower relative abundance of Proteobacteria (23.2 vs. 29.4%, *P* < 0.001) and higher abundance of Firmicutes (55.5 vs. 42.9%, *P* < 0.001) in the LBW group compared to NBW group. At the phylum level (Figure 3B), the gut microbiota of the NBW group contained a greater relative abundance of Proteobacteria at stages 1 and 3 (*P* = 0.003 and *P* < 0.001, respectively), while the LBW group had a greater abundance of Firmicutes at stages 1, 3, and 4 (all *P* < 0.01). At the family level, the gut microbiota of the LBW group had a greater abundance of *Ruminococcaceae* at stages 3 to 5 (all *P* < 0.05). At the genus level, there were a large number of genera with significant different abundances between the two groups (adjusted *P* < 0.01, Wilcoxon rank sum test corrected for multiple testing by the Benjamini and Hochberg method; Figure 3C). The gut microbiota of the NBW group contained a greater relative abundance of *Haemophilus* throughout the first 3 months (all *P* < 0.05) except stage 2, and an unknown *Enterobacteriaceae* genus at stages 3 to 5 (all *P* < 0.05), while the LBW group had a greater abundance of *Enterococcus* throughout the first 3 months (all *P* < 0.05), and *Streptococcus* at stages 2 and 3 (both *P* < 0.05), as well as *Klebsiella* at stages 4 and 5 (both *P* < 0.01) (Figure 3D,E).

**FIGURE 4:**
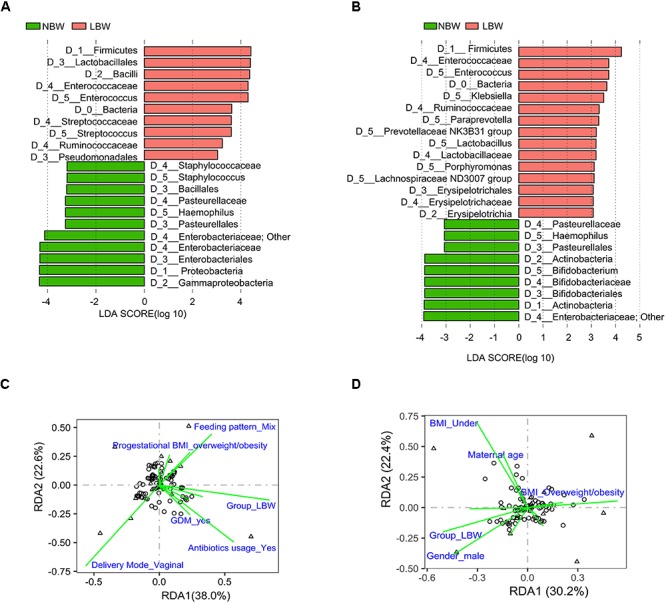
Influencing factors affected the infant gut microbiota composition at stages 3 and 4. Results of LEfSe on gut microbiota of infants at stage 3 **(A)** and stage 4 **(B)**. RDA showing the factors associated with microbiota composition at stage 3 **(C)** and stage 4 **(D)**. Influencing factors that significantly (*P* < 0.05) explain the variations are shown. LBW, low birth weight; NBW, normal birth weight; LEfSe, linear discriminant analysis effect size; RDA, redundancy analysis.

To explore the variation of the microbial community composition between the NBW and LBW infants, we performed LEfSe tests to detect differences in relative abundance of bacterial taxa across the two stages (Figure 4A,B). Taxa which were enriched in the NBW group included Proteobacteria (LDA = 4.339, *P* < 0.001) phylum at stage 3, an unknown *Enterobacteriaceae* genus (LDA = 4.125, *P* < 0.001 and LDA = 3.919, *P* = 0.021), and *Haemophilus* (LDA = 3.261, *P* = 0.010 and LDA = 3.066, *P* < 0.001) genus at stages 3 and 4. It was noteworthy that *Bifidobacterium* (LDA = 3.883, *P* = 0.036) was enriched in NBW infants at stage 4. Conversely, taxa enriched in the LBW infants included Firmicutes (LDA = 4.418, *P* < 0.001 and LDA = 4.240, *P* = 0.003) phylum, *Ruminococcaceae* (LDA = 3.236, *P* = 0.007 and LDA = 3.321, *P* = 0.020) family, *Enterococcus* (LDA = 4.229, *P* < 0.001 and LDA = 3.718, *P* = 0.016) genus at stages 3 and 4, *Streptococcus* (LDA = 3.605, *P* = 0.045) genus at stage 3, *Lachnospiraceae ND3007 group* (LDA = 3.111, *P* = 0.004) and *Klebsiella* (LDA = 3.605, *P* = 0.045) genus at stage 4.

### Influencing Factors Affected the Infant Gut Microbiota Composition

The differences in microbiota development over time were further explored via RDA throughout all stages. Maternal factors including age, pregestational BMI, status of GDM, gestational hypertension, as well as delivery mode, birth weight, gender and gestational age of infants were taken into consideration at stage 1. Besides the above factors, antibiotic usage, hospital stay and type of feeding were taken into consideration at other stages. Birth weight was the only factor significantly explaining the observed variation in microbiota composition between samples at stages 1 (3.22%, *P* = 0.026) (Supplementary Table 6) and 5 (3.58%, *P* = 0.026). At stage 3, it still was the main factor explaining the observed variation (7.55%, *P* < 0.001), while other factors were delivery mode (4.78%, *P* = 0.002) and antibiotic usage (3.50%, *P* = 0.003) (Figure 4C and Supplementary Table 7). The observed variation could be explained by birth weight (3.40%, *P* = 0.004) and pre-gestational BMI (4.61%, *P* = 0.003) at stage 4 (Figure 4D and Supplementary Table 8). Based on whether antibiotics was used at stage 3, the LBW group was divided into two subgroups. There were significant differences in bacterial compositions between the two subgroups (adonis *P* = 0.01), as well as compared with the NBW infants, respectively (both adonis *P* < 0.001) (Supplementary Table 9). LEfSe tests showed that *Staphylococcus* (LDA = 3.171, *P* = 0.001) was enriched in LBW infants who received no antibiotics, while *Enterococcus* (LDA = 4.504, *P* < 0.001) was enriched in LBW infants who received antibiotics treatment at stage 3 (Supplementary Figure 1).

## Discussion

In our study, we characterized gut microbiota profiles of the LBW and NBW infants, described their change trend across different stages within each group, identified the differences between the two groups, and explored the underlying factors responsible for microbiota community in the first 3 months of early life, using data from a prospective cohort study on Chinese infants.

Low birth weight infants have an underdeveloped immune system and a slower progression of bacterial colonization (Keunen et al., 2015), possibly resulting in a different diversity and composition of gut microbiota, which was supported by the results in our study from birth (stage 1) to 3 months of early life (stage 5). Gut microbiota disturbance often occurs before the onset of some infectious diseases, and changes in gut microbiota could be considered as an early predictor of infant sepsis (Madan et al., 2012). It is widely believed that disturbance in gut microbiota is one of the main causes of NEC and late-onset sepsis (Young et al., 2017). In this study, we found that intra- and inter-individual diversity differed by birth weight throughout the 3 months of early life, indicating that LBW infants might have a distinct gut microbiota compared with NBW infants during the early postnatal period. Alpha-diversity of both groups displayed a decreasing trend followed by slight variations. It was noteworthy that alpha-diversity of gut microbiota changed drastically in NBW infants at the early stages (stages 1 and 2) while the difference between these stages was not significant in LBW infants. A larger inter-individual variation of alpha-diversity was observed in LBW infants, particularly at stage 2. Considering the fact that all of the LBW infants were hospitalized in NICU immediately after birth, whereas the NBW infants were discharged together with their mothers, it could be explained by the fact that the environmental microbiota of NICU is relative stable and has lower bacterial abundances than other less controlled environments, so that microbe exposure of LBW infants is reduced.

Our study showed that the LBW group had a greater abundance of *Enterococcus* throughout all the stages, which is consistent with previous research (Moles et al., 2015; Raveh-Sadka et al., 2016). A recent study showed that health care-associated infections caused by *Enterococcus* are increasing (Pidot et al., 2018). The researchers tested alcohol tolerance of 139 hospital isolates of *Enterococcus* obtained between 1997 and 2015, and found that *Enterococcus* isolates after 2010 were 10-fold more resistant to killing by alcohol-based disinfectants than were older isolates (Pidot et al., 2018). The greater abundance of *Enterococcus* in LBW infants might be associated with the hospital stay in their early life. After hospital discharge, a major obstacle for the microbiota development among the LBW infants was the overgrowth of *Enterococcus*, which might inhibit the normal succession throughout the first 3 months of life. This is consistent with the research of Korpela et al. (2018), which showed that the development of microbiota in premature infants was disrupted by persistently increased abundance of *Enterococcus.* Zwittink et al. (2018) reported that community richness and diversity were associated with the dominance of specific bacterial taxa such as *Enterococcus*, leading to differences in microbial networks. This higher abundance of *Enterococcus* might increase a health risk for the LBW infants, as some *Enterococcus* species emerged from gut commensals to nosocomial pathogens via the acquisition of multi-drug resistance and other virulence determinants (Arias and Murray, 2012). Our study showed that the differences of microbiota diversity and composition of the two groups still existed at 3 months of life, which is consistent with most previous studies (Dahl et al., 2018). We also found that *Klebsiella*, enriched in the LBW group at stages 4 and 5, and which is frequently implicated in nosocomial infection (Brooks et al., 2017), might be acquired from medical procedures. Beyond nutritional components, breast milk contains some important bioactive substances such as microbes, oligosaccharides, cytokines, immunoglobulins, and proteins, which directly influence the development of infants and shape their intestinal microbiota colonization (Agostoni et al., 2010). Those bioactive substances are considered not only protective but also stimulate the development and maturation of the immature immune system (Ballard and Morrow, 2013). Our results have demonstrated that the samples from the NBW infants showed a significant greater presence of *Bifidobacterium* with the capacity to digest human milk oligosaccharides at stage 4 and that might promote the specific lipopolysaccharide signaling and its contribution to the burgeoning immune system in infancy (Parra-Llorca et al., 2018). Our results are consistent with the research of Stewart (Stewart et al., 2017), which showed that *Bifidobacteria* was beneficial to preterm infants, and its presence may directly protect against gut epithelial translocation.

Microbiota of meconium might be acquired *in utero* or shaped by delivery mode (Dominguez-Bello et al., 2010), which is quite different from subsequent infant depositions. We analyzed the association of gut microbiota with maternal and neonatal factors, and found that birth weight was the only factor significantly explaining the observed variation in microbiota composition of meconium. In our study, all meconium samples were obtained on the first day of life; thus, factors including delivery mode and diet should not affect our detection of microbial biomarkers. This is expected because early intestinal microbiota colonization of infants can already occur *in utero* from maternal microbiota, which are not affected by factors at birth, such as delivery mode and type of feeding (Neu and Walker, 2011). Antibiotic exposure influences the establishment of gut microbiota in LBW infants, resulting in the drastic change of its diversity and richness (Bäckhed et al., 2015; O’Connor et al., 2016). Our study showed that antibiotic treatment was a main factor significantly explaining the observed variation in microbiota composition of the samples at stage 3. This could explain why we observed absence of pathogens including *Staphylococcus* and *Haemophilus* in LBW infants, who were more likely to get antibiotic treatment. It was also notable that *Staphylococcus* enriched in LBW infants who received no antibiotics at stage 3, which was coincident with NBW infants, indicating that the antibiotic treatment might strongly influence the abundance of *Staphylococcus*. There were significant differences in bacterial compositions between NBW infants and LBW infants receiving antibiotic treatment or not, which illustrated that antibiotic usage also might play a role in gut microbiota establishment, but is not the most critical factor. Delivery mode was another main factor that could explain the variation of gut microbiota at 2 weeks after birth, but not at birth. Dominguez-Bello et al. (2010) suggested that incidental exposures to skin bacteria could contribute to the microbiota of C-section delivered infants, while the vaginally delivered infants acquired bacterial communities resembling their own mother’s vaginal microbiota. Our results have also demonstrated that the pregestational BMI has an impact on gut microbial composition of infants and the LBW group had a greater abundance of *Lachnospiraceae ND3007 group* at stage 4. This is consistent with the research of Tun et al. (2018), which showed that maternal weight status shape early-life gut microbial development of offspring, and the intergenerational transmission of overweight or obesity is mediated by a higher abundance of *Lachnospiraceae*.

The limitation of this study is that we analyzed time series data in relation to gestational age rather than postconceptional age. Postconceptional age is often used in some studies based on the fact that preterm infants are often considered to reach a physiological maturation similar to that in term infants when they reach term-corrected postconceptional age. However, based on the corrected age for preterm infants, the comparison of the gut microbiota between LBW and NBW infants at different stages may not be possible for the following reason: the GIT of the fetus is nearly sterile but becomes rapidly colonized in the early days of life. It is influenced by factors such as the mode of delivery, the maternal microbiota, milk source and the surrounding environment. So, in the preterm infant the microbiota will have been influenced by external factors for a longer period of time than the term infant of same postconceptional age. Therefore, in our present study, we collected fecal samples according to specific postnatal time points and considered gestational age as an influencing factor, rather than using postconceptional age. We did not collect information about intrapartum antibiotics use, types of formula to which infants were exposed, samples of NICU, and the physical environment and thus could not determine their contribution to gut microbiota of infants in our study. The number of infants fed with formula included in this study was limited (*n* = 3) because it was difficult to recruit infants with formula feeding and randomization on feeding practices was not ethically acceptable. In addition, the amount of formula intake of mixed feeding practice was hard to record as it varied with the mother’s milk supply and the LBW infant’s demand. Therefore, the effect of formula and mix feeding practices on the development of gut microbiota could not be measured precisely. A multi-center study should be considered as the gut microbial community differed in the study locations such as different hospitals (Ravi et al., 2017) as well as rural or urban environments (Lehtimäki et al., 2017).

In summary, our findings showed that the gut microbial community differed in LBW and NBW infants from birth to 3 months of their early life. We found birth weight to be an important influencing factor for the microbial community of infants, as well as delivery mode, antibiotic usage, and pregestational BMI of mothers.

## Ethics Statement

The study was approved by the Ethics Committee of the Beijing Obstetrics and Gynecology Hospital (No. 2017-KY-027-01), and informed written consent was obtained from parents of each infant.

## Author Contributions

CY, BZ, and YX designed the study. CC, RL, YW, XD, HZ, GL, QS, XH, LC, TJ, and JZ recruited the infants and collected the data and samples. YX, NL, and YH performed the microbiological analyses. YX and YH analyzed the data. CC and YX generated the figures and wrote the manuscript. JS and NB critically reviewed and edited the manuscript. All authors agreed to be accountable for all aspects of the work in ensuring that questions related to the accuracy or integrity of any part of the work are appropriately investigated and resolved.

## Conflict of Interest Statement

The authors declare that the research was conducted in the absence of any commercial or financial relationships that could be construed as a potential conflict of interest.

## Abbreviations

BMI: body mass index
C: cesarean
GDM: gestational diabetes mellitus
LBW: low birth weight
LEfSe: linear discriminant analysis effect size
NBW: normal birth weight
NICU: neonatal intensive care unit
PCoA: principal coordinate analysis
PCR: polymerase chain reaction
RDA: redundancy analysis
RT: room temperature.
