# Supplementary material for: Longitudinal Gut Bacterial Colonization and Its Influencing Factors of Low Birth Weight Infants During the First 3 Months of Life
Source: Front Microbiol. 2019 May 15;10:1105. doi: 10.3389/fmicb.2019.01105 (PMC6529567; doi:10.3389/fmicb.2019.01105)

***Supplementary Material***

**Longitudinal gut bacterial colonization and its influencing factors of low birth weight infants during the first 3 months of life**

Cheng Chi*, Yong Xue*, Na Lv, Yanan Hao, Ruixia Liu, Yanxin Wang, Xin Ding, Huihui Zeng, Geng Li, Qun Shen, Xiaosong Hu, Lijun Chen, Tiemin Jiang, Junying Zhao, Nicholas Buys, Jing Sun, Chenghong Yin, Baoli Zhu

*These authors contribute equally to this work.

Corresponding Author:

Chenghong Yin, modscn@126.com

Baoli Zhu, [zhubaoli@im.ac.cn](mailto:zhubaoli@im.ac.cn)

Jing Sun, j.sun@griffith.edu.au

**Legends:**

**Supplementary Table 1** Clinical data of the study participants.

**Supplementary Table 2** *P*-value of alpha-diversity measured by Shannon and Chao1 index between stages.

**Supplementary Table 3** *P*-value of alpha-diversity measured by Shannon and Chao1 index between NBW and LBW groups.

**Supplementary Table 4** PERMANOVA scores on Bray-Curtis distance beta-diversity between NBW and LBW groups.

**Supplementary Table 5** PERMANOVA scores on Bray-Curtis distance beta-diversity between term NBW and preterm LBW infants.

**Supplementary Table 6** PERMANOVA scores of influencing factors effects on Bray-Curtis distance β-diversity at stage 1.

**Supplementary Table 7** PERMANOVA scores of influencing factors effects on Bray-Curtis distance β-diversity at stage 3.

**Supplementary Table 8** PERMANOVA scores of influencing factors effects on Bray-Curtis distance β-diversity at stage 4.

**Supplementary Table 9** PERMANOVA scores on Bray-Curtis distance beta-diversity between NBW and LBW infants received antibiotic treatment or not at stage 3.

**Supplementary Figure 1** Results of LEfSe on gut microbiota of LBW infants received antibiotic treatment or not at stage 3

| **Supplementary Table 1** Clinical data of the study participants. | | | | | | | | | | | | |
| --- | --- | --- | --- | --- | --- | --- | --- | --- | --- | --- | --- | --- |
| ID | Group | GDM | GestationalHypertension | Gender | BirthMode | GA | BirthWeight | AntibioticsStage3 | FeedingStage3 | HospitalizedStage3 | MaternalAge | ProgestationalBMI |
| FC102 | NBW | no | no | male | C-section | 39 | 3640 | no | BreastMilk | no | 33 | 23.52 |
| FC103 | NBW | no | no | male | C-section | 38 | 2865 | no | BreastMilk | no | 30 | 19.05 |
| FC104 | NBW | no | no | male | C-section | 39 | 3585 | no | Mix | no | 37 | NA |
| FC105 | NBW | no | no | male | C-section | 38 | 3355 | no | BreastMilk | no | 32 | NA |
| FC107 | NBW | no | no | female | Vaginal | 39 | 3250 | no | BreastMilk | no | 33 | 20.03 |
| FC108 | NBW | no | no | male | C-section | 38 | 3380 | no | BreastMilk | no | 46 | 22.66 |
| FC109 | NBW | no | no | female | Vaginal | 39 | 3620 | no | BreastMilk | no | 38 | 20.83 |
| FC110 | NBW | no | no | male | C-section | 38 | 3965 | no | BreastMilk | no | 32 | 24.03 |
| FC111 | NBW | yes | no | female | C-section | 38 | 3310 | no | Mix | no | 37 | 29.38 |
| FC112 | NBW | no | no | female | Vaginal | 38 | 2810 | no | BreastMilk | no | 40 | 21.72 |
| FC114 | NBW | no | no | male | Vaginal | 39 | 3400 | no | Mix | no | 28 | 22.49 |
| FC117 | NBW | no | no | male | C-section | 38 | 2700 | yes | BreastMilk | no | 32 | 18.82 |
| FC118 | NBW | no | no | female | C-section | 40 | 3780 | no | BreastMilk | no | 28 | 22.58 |
| FC119 | NBW | no | no | female | Vaginal | 40 | 3300 | no | BreastMilk | no | 34 | NA |
| FC120 | NBW | no | no | female | Vaginal | 40 | 2910 | no | BreastMilk | no | 29 | 24.34 |
| FC122 | NBW | no | no | male | C-section | 36 | 2800 | no | NA | no | 34 | NA |
| FC123 | NBW | no | no | female | C-section | 37 | 2865 | no | Mix | no | 33 | 23.42 |
| FC124 | NBW | yes | no | male | Vaginal | 40 | 3570 | no | BreastMilk | no | 31 | 23.62 |
| FC125 | NBW | no | no | male | Vaginal | 38 | 3400 | no | BreastMilk | no | 30 | 24.03 |
| FC127 | NBW | no | no | male | C-section | 38 | 3340 | no | BreastMilk | no | 43 | 18.13 |
| FC128 | NBW | no | no | female | C-section | 38 | 3285 | no | Mix | no | 28 | 21.63 |
| FC129 | NBW | no | no | female | C-section | 39 | 4085 | no | BreastMilk | no | 38 | 20 |
| FC130 | NBW | no | no | female | Vaginal | 38 | 3130 | no | BreastMilk | no | 38 | 25.71 |
| FC131 | NBW | no | no | male | Vaginal | 40 | 3250 | no | BreastMilk | no | 35 | 20.32 |
| FC132 | NBW | no | no | male | C-section | 39 | 3380 | no | BreastMilk | no | 26 | 18.81 |
| FC135 | NBW | no | no | female | C-section | 39 | 3085 | no | BreastMilk | no | 30 | 18.82 |
| FC136 | NBW | no | no | female | C-section | 39 | 3365 | no | Mix | no | 33 | 21.34 |
| FC140 | NBW | no | no | female | C-section | 38 | 3390 | no | BreastMilk | no | 33 | 24.77 |
| FC141 | NBW | no | no | male | C-section | 39 | 3710 | no | Mix | no | 26 | 23.31 |
| FC143 | NBW | yes | no | male | Vaginal | 39 | 3580 | no | NA | no | 34 | NA |
| FC144 | NBW | no | no | female | Vaginal | 38 | 3810 | no | BreastMilk | no | 40 | 23.14 |
| FC145 | NBW | no | no | female | C-section | 37 | 3095 | no | Mix | no | 32 | 24.09 |
| FC146 | NBW | yes | no | female | Vaginal | 39 | 3270 | no | Mix | no | 35 | 27.15 |
| FC147 | NBW | no | no | male | Vaginal | 40 | 3110 | yes | BreastMilk | no | 31 | 18.36 |
| FC148 | NBW | no | no | female | C-section | 39 | 3570 | no | BreastMilk | no | 43 | 21.77 |
| FC149 | NBW | no | no | female | C-section | 39 | 3080 | no | BreastMilk | no | 0 | NA |
| FC152 | NBW | no | no | male | Vaginal | 39 | 3500 | no | Mix | no | 30 | 23.44 |
| FC153 | NBW | no | no | female | Vaginal | 40 | 3380 | no | BreastMilk | no | 33 | 21.03 |
| FC154 | NBW | no | no | male | C-section | 38 | 3560 | no | Mix | no | 35 | 25.39 |
| FC158 | NBW | no | no | male | Vaginal | 37 | 3290 | yes | BreastMilk | no | 29 | 19.6 |
| FC159 | NBW | no | no | female | Vaginal | 41 | 3730 | no | BreastMilk | no | 32 | 24.22 |
| FC160 | NBW | no | no | male | Vaginal | 39 | 2880 | no | BreastMilk | no | 27 | 19.14 |
| FC166 | NBW | no | no | male | C-section | 37 | 2910 | no | BreastMilk | no | 36 | 19.14 |
| FC167 | NBW | no | no | female | Vaginal | 39 | 3000 | no | BreastMilk | no | 38 | 21.36 |
| FC168 | NBW | no | no | male | Vaginal | 37 | 3110 | no | Mix | no | 37 | 22.31 |
| FC169 | NBW | no | no | male | Vaginal | 39 | 3330 | no | BreastMilk | no | 30 | 22.19 |
| FC170 | NBW | no | no | female | C-section | 39 | 3875 | no | Mix | no | 36 | 26.67 |
| FC171 | NBW | no | no | female | C-section | 38 | 3290 | no | NA | no | 36 | 28.91 |
| FC177 | NBW | no | no | male | C-section | 38 | 2620 | no | Mix | no | 39 | 21.09 |
| FC178 | NBW | yes | no | male | C-section | 37 | 3095 | no | Mix | no | 32 | 27.34 |
| FC180 | NBW | no | no | female | Vaginal | 38 | 2660 | no | BreastMilk | no | 30 | 20.81 |
| FC182 | NBW | no | no | female | C-section | 37 | 3590 | no | BreastMilk | no | 37 | 20.51 |
| FC184 | NBW | no | no | female | Vaginal | 39 | 3270 | no | BreastMilk | no | 28 | 18.75 |
| FC185 | NBW | no | no | female | C-section | 37 | 2505 | no | BreastMilk | no | 29 | 26.35 |
| FC186 | NBW | no | no | female | Vaginal | 41 | 3640 | no | Mix | no | 30 | 23.05 |
| FC187 | NBW | no | no | female | C-section | 38 | 3270 | yes | Mix | no | 30 | 19.89 |
| FC188 | NBW | no | no | female | C-section | 38 | 2925 | no | BreastMilk | no | 33 | 20.94 |
| FC189 | NBW | no | no | male | C-section | 38 | 2695 | no | Mix | no | 36 | 20.83 |
| FC191 | NBW | no | no | male | C-section | 38 | 3865 | no | BreastMilk | no | 42 | 28.34 |
| FC192 | NBW | yes | no | male | Vaginal | 40 | 3470 | no | BreastMilk | no | 28 | 23.39 |
| FC193 | NBW | no | no | female | Vaginal | 40 | 3370 | no | Mix | no | 31 | 18.78 |
| FC194 | NBW | no | no | female | Vaginal | 37 | 2910 | no | BreastMilk | no | 39 | 25.71 |
| FC195 | NBW | no | no | male | C-section | 36 | 2800 | no | Mix | no | 31 | 21.45 |
| FC196 | NBW | no | yes | male | C-section | 35 | 2775 | no | Mix | no | 29 | 20.28 |
| FC198 | NBW | no | no | male | C-section | 38 | 2640 | no | Mix | no | 30 | 19.13 |
| FC203 | LBW | no | yes | female | C-section | 35 | 2365 | yes | BreastMilk | no | 39 | 30.44 |
| FC206 | LBW | yes | no | male | C-section | 36 | 1920 | yes | Mix | yes | 35 | NA |
| FC207 | LBW | no | no | female | C-section | 36 | 2390 | yes | NA | yes | 28 | 19.28 |
| FC208 | LBW | no | no | male | Vaginal | 38 | 2330 | no | Mix | no | 36 | NA |
| FC210 | LBW | yes | no | female | C-section | 36 | 2400 | no | Mix | no | 34 | 18.65 |
| FC211 | LBW | yes | no | female | C-section | 36 | 2725 | no | Mix | no | 34 | 18.65 |
| FC212 | LBW | no | yes | female | C-section | 35 | 1735 | yes | Formula | yes | 36 | 22.21 |
| FC213 | LBW | no | no | male | C-section | 37 | 2325 | no | Mix | no | 29 | 26.35 |
| FC214 | LBW | no | no | male | Vaginal | 30 | 2490 | no | Mix | no | 30 | 18.51 |
| FC215 | LBW | no | no | female | C-section | 30 | 1180 | yes | Mix | yes | 33 | 22.66 |
| FC216 | LBW | no | no | female | C-section | 30 | 1250 | yes | Mix | yes | 33 | 22.66 |
| FC217 | LBW | yes | no | female | C-section | 37 | 2420 | yes | Mix | no | 36 | 19.78 |
| FC218 | LBW | yes | no | male | C-section | 37 | 2140 | yes | Mix | no | 36 | 19.78 |
| FC220 | LBW | no | no | female | C-section | 37 | 2290 | yes | BreastMilk | yes | 28 | 18.03 |
| FC221 | LBW | no | no | male | C-section | 31 | 1590 | yes | BreastMilk | yes | 34 | 16.87 |
| FC222 | LBW | no | no | female | C-section | 35 | 2410 | no | Mix | no | 35 | 20.08 |
| FC223 | LBW | no | no | male | Vaginal | 34 | 2280 | yes | Mix | no | 30 | 23.37 |
| FC224 | LBW | no | no | female | C-section | 38 | 2355 | no | NA | no | 36 | NA |
| FC226 | LBW | yes | no | female | C-section | 27 | 1050 | yes | Mix | yes | 29 | 18.59 |
| FC227 | LBW | no | no | female | C-section | 34 | 2530 | yes | Mix | no | 29 | 36.74 |
| FC228 | LBW | no | no | female | C-section | 34 | 1830 | yes | Mix | yes | 29 | 36.74 |
| FC229 | LBW | no | no | male | C-section | 34 | 2395 | no | Formula | no | 22 | 21.97 |
| FC230 | LBW | yes | no | female | Vaginal | 39 | 2470 | no | BreastMilk | no | 34 | 19.53 |
| FC231 | LBW | yes | yes | female | C-section | 28 | 1190 | yes | NA | yes | 40 | NA |
| FC232 | LBW | no | yes | male | C-section | 35 | 2345 | no | Mix | no | 29 | 20.28 |
| FC233 | LBW | no | no | male | C-section | 36 | 2330 | no | Mix | no | 31 | 21.45 |
| FC234 | LBW | no | no | male | C-section | 33 | 1700 | no | BreastMilk | no | 31 | 23.81 |
| FC236 | LBW | no | no | female | C-section | 37 | 2460 | no | Mix | no | 31 | 20.83 |
| FC238 | LBW | no | yes | female | C-section | 37 | 2340 | yes | Mix | no | 34 | 20.81 |
| FC239 | LBW | no | no | female | C-section | 35 | 2400 | yes | Mix | no | 30 | 19.13 |
| FC240 | LBW | no | yes | female | C-section | 38 | 2235 | no | Mix | no | 29 | 23.14 |
| FC241 | LBW | no | yes | male | C-section | 36 | 2300 | no | Mix | no | 29 | 23.14 |
| FC242 | LBW | no | no | male | C-section | 36 | 2330 | no | Mix | no | 30 | 19.22 |
| FC243 | LBW | no | no | female | C-section | 37 | 2455 | no | Mix | no | 30 | 19.22 |
| FC244 | LBW | no | no | male | C-section | 33 | 1950 | yes | Mix | yes | 28 | 16.82 |
| FC245 | LBW | no | no | male | C-section | 33 | 1900 | no | Mix | no | 28 | 16.82 |
| FC246 | LBW | no | no | male | Vaginal | 36 | 2410 | no | Formula | no | 36 | 21.48 |
| FC247 | LBW | yes | yes | female | Vaginal | 35 | 2425 | no | Mix | no | 39 | 19.53 |
| FC248 | LBW | yes | yes | female | Vaginal | 35 | 1665 | yes | Mix | yes | 39 | 19.53 |
| FC249 | LBW | no | no | male | Vaginal | 36 | 2240 | no | BreastMilk | no | 28 | 22.86 |
| FC250 | LBW | no | no | male | C-section | 33 | 1890 | yes | Mix | yes | 31 | 23.98 |
| FC253 | LBW | no | no | female | C-section | 29 | 1385 | yes | Mix | yes | 32 | 34.02 |
| FC254 | LBW | no | no | male | C-section | 29 | 1160 | yes | Mix | yes | 30 | 21.37 |
| FC255 | LBW | no | no | male | Vaginal | 33 | 2120 | yes | Mix | no | 31 | NA |
| FC256 | LBW | no | no | female | C-section | 36 | 2310 | no | Mix | no | 34 | 17.06 |
| FC258 | LBW | yes | yes | male | C-section | 29 | 1040 | yes | NA | yes | 30 | 22.89 |
| FC259 | LBW | no | no | male | C-section | 36 | 2275 | no | Mix | no | 31 | 25.34 |
| FC260 | LBW | no | no | male | C-section | 36 | 2460 | no | Mix | no | 31 | 25.34 |
| FC261 | LBW | no | yes | male | Vaginal | 34 | 2320 | yes | BreastMilk | yes | 26 | 20.2 |
| FC262 | LBW | no | no | female | C-section | 37 | 2180 | yes | NA | yes | 32 | NA |
| FC263 | LBW | no | yes | female | Vaginal | 36 | 2050 | no | Mix | no | 36 | 24.17 |
| FC265 | LBW | no | yes | female | C-section | 32 | 2050 | no | BreastMilk | yes | 32 | 25.39 |
| FC267 | LBW | no | no | male | C-section | 33 | 1650 | yes | BreastMilk | yes | 38 | 20.51 |
| FC268 | LBW | no | no | female | Vaginal | 37 | 2260 | no | Mix | no | 33 | 18.29 |
| FC269 | LBW | no | no | male | Vaginal | 38 | 2005 | yes | Mix | no | 29 | 20.61 |
| FC270 | LBW | yes | no | female | C-section | 30 | 1615 | yes | Mix | yes | 37 | 21.67 |
| FC271 | LBW | yes | no | female | C-section | 30 | 1380 | yes | Mix | yes | 37 | 21.67 |
| FC272 | LBW | no | no | female | Vaginal | 38 | 2170 | yes | Mix | yes | 27 | NA |
| FC273 | LBW | no | no | female | C-section | 37 | 2215 | yes | Mix | no | 29 | 22.21 |
| FC274 | LBW | no | no | male | C-section | 33 | 2050 | yes | Mix | yes | 29 | 25.78 |
| FC275 | LBW | no | no | female | C-section | 37 | 2305 | no | Mix | no | 30 | 22.04 |
| FC276 | LBW | no | no | female | C-section | 37 | 2275 | no | Mix | no | 30 | 22.04 |
| FC278 | LBW | no | no | female | C-section | 34 | 1860 | no | BreastMilk | yes | 36 | 20.9 |
| FC279 | LBW | yes | no | male | Vaginal | 34 | 2090 | yes | NA | yes | 30 | NA |
| FC280 | LBW | no | yes | male | C-section | 27 | 885 | yes | NA | yes | 31 | 22.06 |
| FC281 | LBW | no | no | female | C-section | 36 | 2145 | yes | NA | yes | 28 | NA |
| FC282 | LBW | yes | no | male | C-section | 40 | 2285 | yes | Mix | no | 35 | 28.52 |
| FC283 | LBW | no | no | female | C-section | 36 | 2255 | no | NA | no | 36 | NA |
| FC302 | LBW | no | no | male | C-section | 36 | 2150 | no | Mix | no | 36 | 19.2 |
|  |  |  |  |  |  |  |  |  |  |  |  |  |

**Supplementary Table 2** *P*-value of alpha-diversity measured by Shannon and Chao1 index between stages.

|  | Chao1 | | Shannon | |
| --- | --- | --- | --- | --- |
|  | NBW group | LBW group | NBW group | LBW group |
| S1 vs S2 | < 0.001 | 0.804 | < 0.001 | 1 |
| S1 vs S3 | < 0.001 | 0 | < 0.001 | 0.109 |
| S1 vs S4 | < 0.001 | 0 | < 0.001 | 0.175 |
| S1 vs S5 | < 0.001 | 0.024 | < 0.001 | 1 |
| S2 vs S3 | < 0.001 | 0.032 | 1 | 0.583 |
| S2 vs S4 | < 0.001 | 0.020 | 1 | 0.811 |
| S2 vs S5 | 0.002 | 0.804 | 1 | 1 |
| S3 vs S4 | 1 | 1 | 0.888 | 1 |
| S3 vs S5 | 0.327 | 1 | 0.095 | 1 |
| S4 vs S5 | 1 | 1 | 1 | 1 |

**Supplementary Table 3** *P*-value of alpha-diversity measured by Shannon and Chao1 index between NBW and LBW groups.

|  | Chao1 | Shannon |
| --- | --- | --- |
| S1 | 0.166 | 0.041 |
| S2 | 0.291 | 0.032 |
| S3 | 0.001 | 0.014 |
| S4 | 0.116 | 0.427 |
| S5 | 0.182 | 0.410 |

**Supplementary Table 4** PERMANOVA scores on Bray-Curtis distance beta-diversity between NBW and LBW groups.

| Stage | Df | Sum Sq | Mean Sq | F. Model | R^2^ | *P* |
| --- | --- | --- | --- | --- | --- | --- |
| S1 | 1 | 0.6975 | 0.69745 | 2.6353 | 0.03007 | 0.023 |
| S2 | 1 | 0.6517 | 0.65171 | 1.9063 | 0.02193 | 0.054 |
| S3 | 1 | 2.2408 | 2.24084 | 7.7456 | 0.08175 | 0.001 |
| S4 | 1 | 0.8205 | 0.8205 | 2.9127 | 0.03691 | 0.006 |
| S5 | 1 | 0.6073 | 0.60725 | 2.3781 | 0.03752 | 0.02 |

**Supplementary Table 5** PERMANOVA scores on Bray-Curtis distance beta-diversity between term NBW and preterm LBW infants.

| Stage | Df | Sum Sq | Mean Sq | F. Model | R^2^ | *P* |
| --- | --- | --- | --- | --- | --- | --- |
| S1 | 1 | 0.6167 | 0.61675 | 2.3428 | 0.03151 | 0.018 |
| S2 | 1 | 0.4896 | 0.48955 | 1.3774 | 0.01852 | 0.176 |
| S3 | 1 | 2.2853 | 2.28529 | 7.8889 | 0.09404 | 0.001 |
| S4 | 1 | 0.8163 | 0.81626 | 2.9019 | 0.04151 | 0.004 |
| S5 | 1 | 0.676 | 0.67602 | 2.7598 | 0.04862 | 0.009 |

**Supplementary Table 6** PERMANOVA scores of influencing factors effects on Bray-Curtis distance β-diversity at stage 1.

| Factor | Df | Sum Sq | Mean Sq | F. Model | R^2^ | *P* |
| --- | --- | --- | --- | --- | --- | --- |
| Birth weight | 1 | 0.6518 | 0.65181 | 2.49232 | 0.03218 | 0.026 |
| GDM | 1 | 0.2888 | 0.28880 | 1.10430 | 0.01426 | 0.365 |
| Gestational hypertension | 1 | 0.2504 | 0.25042 | 0.95755 | 0.01236 | 0.418 |
| Gender | 1 | 0.4254 | 0.42541 | 1.62664 | 0.02100 | 0.102 |
| Birth mode | 1 | 0.2032 | 0.20317 | 0.77685 | 0.01003 | 0.579 |
| GestSational Age | 1 | 0.0802 | 0.08018 | 0.30657 | 0.00396 | 0.977 |
| Progestational BMI | 2 | 0.5506 | 0.27529 | 1.05264 | 0.02718 | 0.366 |
| Maternal Age | 1 | 0.2827 | 0.28272 | 1.08102 | 0.01396 | 0.348 |
| Residuals | 67 | 17.5223 | 0.26153 |  | 0.86507 |  |
| Total | 76 | 20.2554 |  |  | 1.00000 |  |

**Supplementary Table 7** PERMANOVA scores of influencing factors effects on Bray-Curtis distance β-diversity at stage 3.

| Factor | Df | Sum Sq | Mean Sq | F. Model | R^2^ | *P* |
| --- | --- | --- | --- | --- | --- | --- |
| Birth weight | 1 | 1.8402 | 1.84018 | 6.9367 | 0.07552 | 0.001 |
| GDM | 1 | 0.1539 | 0.15395 | 0.5803 | 0.00632 | 0.815 |
| Gestational hypertension | 1 | 0.4889 | 0.48896 | 1.8428 | 0.02006 | 0.060 |
| Gender | 1 | 0.3736 | 0.37361 | 1.4083 | 0.01533 | 0.181 |
| Birth mode | 1 | 1.1653 | 1.165330 | 4.3928 | 0.04783 | 0.002 |
| GestSational Age | 1 | 0.3110 | 0.3110 | 1.1723 | 0.01276 | 0.302 |
| Progestational BMI | 2 | 0.5160 | 0.25802 | 0.9726 | 0.02118 | 0.492 |
| Maternal Age | 1 | 0.1308 | 0.13079 | 0.4930 | 0.00537 | 0.874 |
| Antibiotic usage | 1 | 0.8532 | 0.85323 | 3.2163 | 0.03502 | 0.003 |
| Feeding | 2 | 0.5064 | 0.25322 | 0.9545 | 0.02079 | 0.499 |
| Hospital Stay | 1 | 0.5178 | 0.51778 | 1.9518 | 0.02125 | 0.052 |
| Residuals | 66 | 17.5086 | 0.26528 |  | 0.71857 |  |
| Total | 79 | 24.3658 |  |  | 1.00000 |  |

**Supplementary Table 8** PERMANOVA scores of influencing factors effects on Bray-Curtis distance β-diversity at stage 4.

| Factor | Df | Sum Sq | Mean Sq | F. Model | R^2^ | *P* |
| --- | --- | --- | --- | --- | --- | --- |
| Birth weight | 1 | 0.7297 | 0.72966 | 2.73462 | 0.03395 | 0.004 |
| GDM | 1 | 0.4016 | 0.40161 | 1.50516 | 0.01869 | 0.133 |
| Gestational hypertension | 1 | 0.2678 | 0.26776 | 1.00352 | 0.01246 | 0.423 |
| Gender | 1 | 0.2661 | 0.26614 | 0.99744 | 0.01238 | 0.448 |
| Birth mode | 1 | 0.1722 | 0.17219 | 0.64532 | 0.00801 | 0.773 |
| Antibiotics Usage | 1 | 0.3836 | 0.38364 | 1.43782 | 0.01785 | 0.157 |
| Progestational BMI | 2 | 0.9910 | 0.49549 | 1.85699 | 0.04611 | 0.025 |
| Maternal Age | 1 | 0.4675 | 0.46755 | 1.75227 | 0.02176 | 0.076 |
| Feeding | 1 | 0.7344 | 0.36719 | 1.37614 | 0.03417 | 0.128 |
| Residuals | 64 | 17.0767 | 0.26682 |  | 0.79461 |  |
| Total | 75 | 21.4906 |  |  | 1.00000 |  |

**Supplementary Table 9** PERMANOVA scores on Bray-Curtis distance beta-diversity between NBW and LBW infants received antibiotic treatment or not at stage 3.

|  | Df | Sum Sq | Mean Sq | F. Model | R^2^ | *P* |
| --- | --- | --- | --- | --- | --- | --- |
| NBW vs. LBW antibiotics no | 1 | 1.1862 | 1.18616 | 7.0575 | 0.09403 | 0.001 |
| NBW vs. LBW antibiotics yes | 1 | 2.4635 | 2.46347 | 15.506 | 0.20536 | 0.001 |
| LBW antibiotics no vs.  LBW antibiotics yes | 1 | 0.6753 | 0.67534 | 3.8874 | 0.09281 | 0.01 |

**Supplementary Figure 1** Results of LEfSe on gut microbiota of LBW infants received antibiotic treatment or not at stage 3.


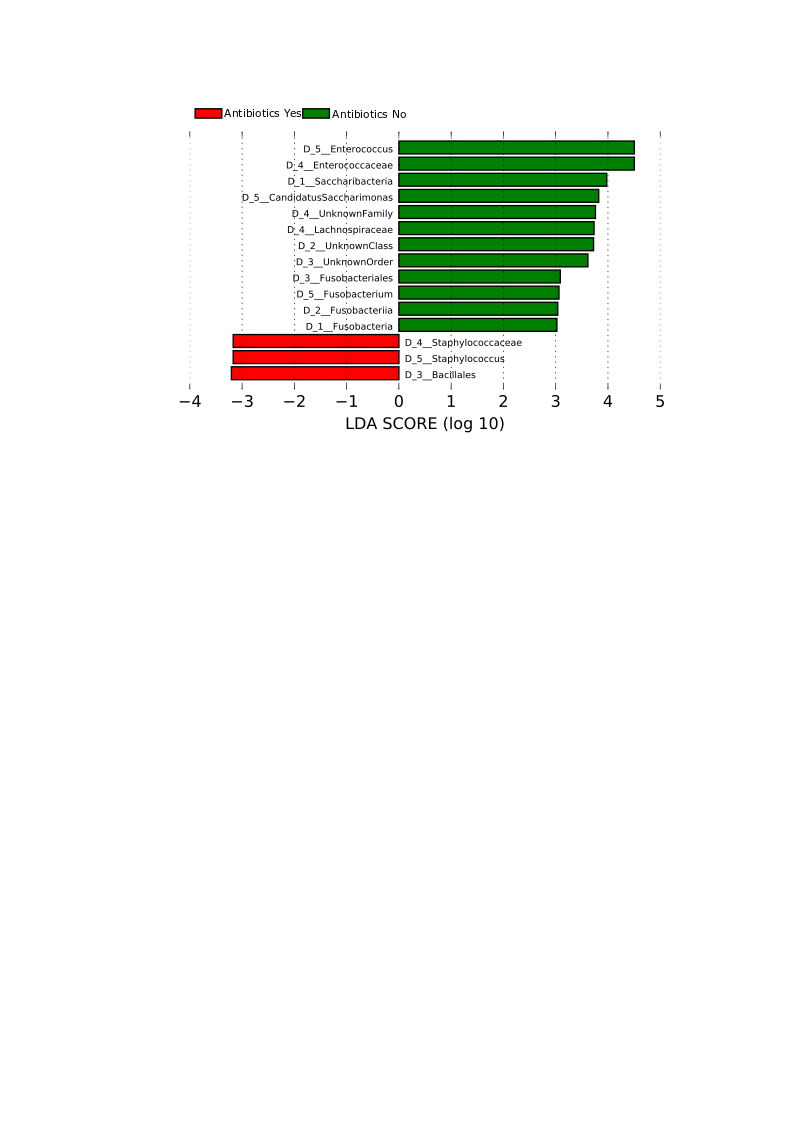

Supplement: Supplementary file 1 [file Table_1.DOCX]
